# Case report: A novel cause of acute liver failure in children: A combination of human herpesvirus‐6 infection and homozygous mutation in *NBAS* gene

**DOI:** 10.1002/jcla.24343

**Published:** 2022-03-29

**Authors:** Ying Cheng, Zhi Xia, Chengjiao Huang, Hui Xu

**Affiliations:** ^1^ Department of Pediatric Intensive Care Unit Maternal and Child Health Hospital of Hubei Province (Women and Children’s Hospital of Hubei Province) Wuhan China

**Keywords:** acute liver failure, human herpesvirus‐6, *NBAS*

## Abstract

Etiologies of acute liver failure in children can be multiple factors including virus infection, drug‐induced damage, and different pathogens. Next‐generation sequencing (NGS) is an emerging method for pan‐pathogen screening. Here we reported a case of acute liver failure in a 15‐month‐old male, using NGS and gene sequencing to determine the cause of acute liver failure may be caused by pathogens, drug‐induction and pathogenic variant gene.

## 1 INTRODUCTION

Acute liver failure in children is a rare life‐threatening disease and has many causes. It is known that neuroblastoma amplified sequence (*NBAS*, NM_015909) gene defects can cause infantile liver failure syndrome‐2 (ILFS‐2). *NBAS* is mapped to chromosome 2p24.3, containing 52 exons. Neuroblastoma‐amplified gene protein (NBAS, NP_056993) encoded by *NBAS* gene includes two leucine zipper domains, a ribosomal protein S14 motif and a Sec39‐like domain, involved in Golgi‐to‐endoplasmic reticulum retrograde transport. The function is proposed to depend on its association in the NRZ complex which is believed to play a role in SNARE assembly at the ER (PubMed: 19369418).^1^ *NBAS* mutations have been found in a multisystem disease affecting the liver, eye, immune system, connective tissue, and bone. In this paper, the clinical data of a child with acute liver failure were analyzed, combined with peripheral lymphocyte subsets analysis, next‐generation sequencing (NGS), gene sequencing, and other laboratory methods, to diagnose ILFS‐2, which can be caused by human herpesvirus‐6 (HHV‐6) infection, drug‐induced, and *NBAS* gene mutation. The current findings may improve our understanding of the molecular mechanism of pediatric acute liver failure.

## 2 CASE PRESENTATION

**FIGURE 1:**
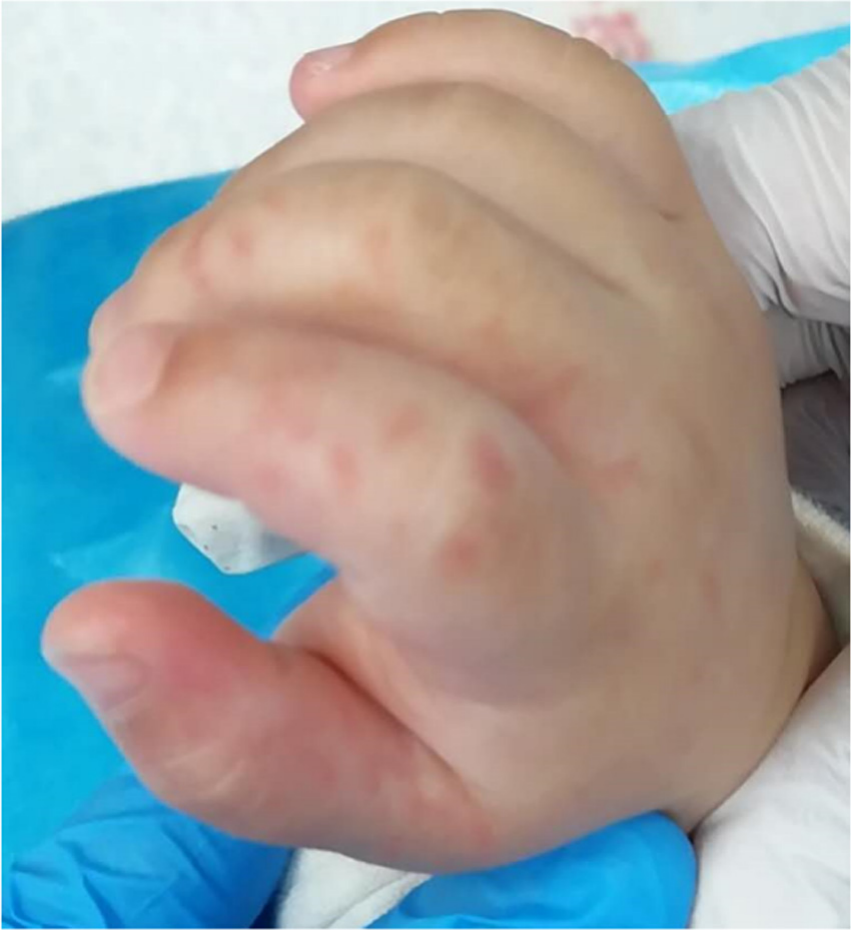
Rashes on the patient's hand

A 15‐month‐old Chinese boy was admitted to the Pediatric Intensive Care Unit (PICU) due to a coma in the night of May, 2020. He had been in poor mental status with fever for 3 days, vomiting for 2 days, and diarrhea with convulsions for 1 day. The boy had a maximum body temperature of 39°C after onset, vomiting, diarrhea, white stools with many white granules 3–4 times a day. The boy took "amoxicillin" and "ibuprofen suspension drops" at home after illness and convulsed 1 time at 21:30 on the day of admission. The symptoms showed upturned eyes, gaze, stiff limbs, and confusion, which lasted for more than 2 h. The emergency physician found red rashes on the both feet and the hands (Figure 1). After the emergency intramuscular injection of midazolam 3 mg, the convulsions gradually eased, but the consciousness was still unclear. The cause of coma was conjectured. Severe hand‐foot‐and‐mouth disease (HFMD) was raised as a differential diagnosis. The patient had been in good health. There were no specific family disease history, or contact history of infectious diseases, or history of food and drug allergies reported in the patient.

On the first day of admission (Day 1), a physical examination revealed the following: the boy's temperature was 37°C; respiratory rate was 35 beats per minute; heart rate was 165 beats per minute; blood pressure was 90/52 mmHg; his peripheral capillary oxygen saturation (SpO_2_) was 88%; and the boy was confused, delirium, and had poor mental response. Several red rashes can be seen on the face, hands, and feet. Pharyngeal hyperemia, no herpes. His abdomen was soft, splenomegaly was not palpated, but hepatomegaly 3 cm below costal margins was palpated. His kerning sign, brudzinkski sign, and babinski sign were all negative.

High incidence of HFMD occurs in Wu Han during annual May. The boy had persistent hyperthermia, combined with gastrointestinal and neurological symptoms, hand and foot rashes. The signs suggested that it might be a severe HFMD. If it was based on previous diagnosis and treatment experience, the boy's condition would deteriorate progressively in the next time. The pediatricians had been prepared well for tracheal intubation for respiratory support at any time to deal with neurogenic pulmonary edema and circulatory failure, which are caused by severe HFMD‐induced brainstem encephalitis.

The laboratory results reported as critical values: aspartate aminotransferase 7642.4 U/L (normal range =15–60); alanine aminotransferase 7179.9 U/L (normal range =13–45) (Table 1); lactate dehydrogenase 11550.7 U/L (normal range =120–300); the coagulation function also reported a critical value: prothrombin time measurement (PT) 114.7s (normal range =9.4–12.5). Blood ammonia (AMM)138.9 μmol/L (normal range =10–47); lactic acid 2.5 mmol/l (normal range =0.5–1.7). At this time, the focus seemed to have suddenly shifted from severe HFMD to various diseases that might cause acute liver failure. Further examination showed that pathogens such as SARS‐CoV‐2, CA16, EV71, hepatitis A, B and C, MP, TP, CMV, EBV, flu virus, and TB, were all negative. The chest computed tomography (CT)scan showed that the boy had pneumonia and fatty liver. The boy's mother denied the family history of liver disease and recent history of unclean eating. After the illness, he was only given "ibuprofen suspension drops" orally for defervescence and "amoxicillin" for antibiotic treatment at home.

**TABLE 1:** Liver function test results

ALT (U/L)	AST (U/L)	LDH (U/L)	PT (s)	AMM (μmol/L)	Lac (mmol/l)
7642.4	7179.9	11550.7	114.7	138.9	2.5

Because the boy was still unconscious, treatment was performed with plasma exchange, which was performed on the Day 1 and Day 3, 2 h each time.

On Day 5, the temperature dropped to normal. His mind was clear and his mental state improved significantly. Rashes subsided. The laboratory results were AST 65.6 U/L, ALT 374.4 U/L, PT 13.6s, and AMM 59.1 μmol/L, cardiac troponin I (cTnI) 0.057 ng/ml (normal range =0–0.04), four items of hepatic fibrosis hyaluronic acid HA > 2300 ng/ml (normal range < 100), laminin LN 852 ng/ml (normal range < 50), type III procollagen N‐terminal peptide PIIIP > 2000 ng/ml (normal range < 30), type IV collagen CIV 271 ng/ml (normal range < 30); 8 items of liver disease autoantibodies were negative. Serum immunoglobulin (Ig) profile and complement was as follows: C3 0.20 g/l (normal range =0.65–1.39), C4 0.05 g/l (normal range =0.16–0.38), IgA, IgG, and IgM normal. The peripheral lymphocyte subsets analysis, assessed by flow cytometry, revealed the following: NK%: 6.31% (normal range =3–16), CD3%: 67.78% (normal range =39–73), CD3/CD8%: 22.44% (normal range =11–32), CD3/CD19%: 22.18% (normal range =7–41), CD4 /CD8 ratio: 1.86 (normal range =0.98–1.94), NK‐cell numbers: 52.26 cells/μl (normal range =100–1400), CD3 cell numbers: 561.51 cells/μl (normal range =1400–8000), CD3/CD4 cell numbers: 346.64 cells/μl (Normal range =900–5500), CD3/CD8#: 186.14 cells/μl (normal range =400–2300), CD3/CD19 cell numbers: 183.57 cells/μl (normal range =600–3100), CD45: 828.49 cells/μl. Five cytokines IL‐6: 20.53 pg/ml (normal range =0–20), IL‐10: 77.09 pg/ml (normal range =0–5.9), IL2, IL4, TNF‐α, IFN‐γ were normal. PCT: 2.48 ng/ml (normal range = <0.1), blood smear: lobulated nuclei 27%, rod‐shaped nuclei 14%, lymphocytes 45%, heteromorphic lymphocyte 12%, monocytes 2%; ferritin > 1500 ng/ml (normal range =23.9–336.2). Urinary organic acid: Phenyllactic acid increases, 4‐hydroxyphenyllactic acid and 4‐hydroxyphenylpyruvate increase, might be secondary to liver damage. EEG showed diffuse slow wave activity. Metagenome Next‐generation sequencing (mNGS)of blood sample detected Human herpesvirus 6B (HHV‐6B) infections. However, the parents did not agree with liver biopsy.

## 3 METHODS AND MATERIALS

### 3.1 Whole exome sequencing

To understand the molecular genetic basis of the family diseases, we performed whole exome sequencing and confirmatory Sanger sequencing of the proband, the proband’ s father and mother. ^2^, ^3^, ^4^

Sample collection. The EDTA‐treated peripheral blood were collected with informed consent of the patient and his healthy parents.

DNA extraction. The whole blood genomic DNA was extracted using the Blood Genome Column Medium Extraction Kit (Kangweishiji) according to the manufactural instructions. The extracted DNA samples were subjected to quality controlling using Qubit 2.0 fluorimeter and electrophoresis with 0.8% agarose gel for further protocol.

Whole exome library construction. Protein‐coding exome enrichment was performed using xGen Exome Research Panel v1.0(IDT) that consists of 429,826 individually synthesized and quality‐controlled probes, which targets 39 Mb protein‐coding region (19,396 genes) of the human genome and covers 51 Mb of end‐to‐end tiled probe space.

Sequencing. High‐throughput sequencing was performed by Illumina NovaSeq 6000 series sequencer (PE150), and not less than 99% of target sequence were sequenced. We confirmed the result by using Sanger sequencing. Primer sequences of the *NBAS* c.3596 (exon31)G>A were as follows: 5′‐ TAAAATAATGGGTCAAGGGCAGGA‐3′ (sense) and 5′‐GGAATGAATTCCTGGGATGTGGTA‐3′ (antisense). The sequencing process was performed by Chigene (Beijing) Translational Medical Research Center Co. Ltd.

### 3.2 Identification of variants

Quality control. Raw data were processed by fastp for adapters removing and low‐quality reads filtering.

Variants calling. The paired‐end reads were performed using Burrows–Wheeler Aligner (BWA) to the Ensemble GRCh37/hg19 reference genome. Base quality score recalibration together with SNP and short indel calling was conducted using GATK. According to the sequence depth and variant quality, SNPs and indels were screened, and high quality and reliable variants were obtained.

Variants annotation and pathogenicity prediction. The online system independently developed by Chigene (www.chigene.org) was used to annotate database‐based minor allele frequencies (MAFs), and ACMG practice guideline‐based pathogenicity of every yielded gene variant, and the system also provide a serial software packages for conservative analysis and protein product structure prediction. The databases for MAFs annotation include 1,000 genomes, dbSNP, ESP, ExAC, and Chigene in‐house MAFs database; Provean, Sift, Polypen2_hdiv, Polypen2_hvar, Mutationtaster, M‐Cap, and Revel software packages were used to predict protein product structure variation. As a prioritized pathogenicity annotation to ACMG guideline, OMIM, HGMD, and ClinVar databases were used as conferences of pathogenicity of every variant. To predict functional change of variants on the splicing sites, MaxEntScan, dbscSNV, and GTAG software packages were used instead of product structure prediction software.

**FIGURE 2:**
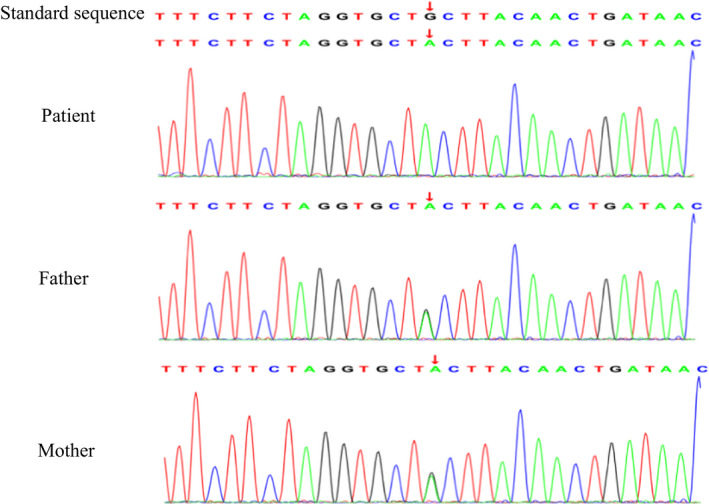
Sanger sequencing of the patient and his parents

Trio WES analysis of this family allowed the identification of a *NBAS* gene homozygous missense variant NM_015909 (*NBAS*): c.3596G>A (p. Cys1199Tyr) in the patient, and heterozygous in his healthy parents, and also confirmed this result by Sanger sequencing (Figure 2). This variant is absent from the publicly available databases (1000 Genomes, NCBI dbSNP, gnomAD), predicted as pathogenic by in silico analysis (Polyphen2, SIFT, mutationtaster, M‐CAP, and REVEL), and also predicted that this variant occurred at conserved position (GERP: 5.73,phyloP: 1.008). In addition, this variant in compound heterozygosity with other *NBAS* variants were reported in multiple liver failure patients.^5^, ^6^ This variant was associated with liver failure.^5^, ^6^ ILFS‐2 is caused by homozygous or compound heterozygous mutation in the *NBAS* gene. It is an autosomal recessive disorder characterized by recurrent episodes of acute liver failure during intercurrent febrile illness. This patient's phenotype is highly specific for this disease. So, variant c.3596G>A was classified as likely pathogenic variant according to the American College of Medical Genetics and Genomics (ACMG) standards and guidelines (2015).

Final diagnosis: (1) *NBAS* gene deficiency disease (Infantile liver failure syndrome‐2); (2) Human herpesvirus 6B (HHV‐6B) infections; (3) Pneumonia.

Treatment and follow‐up visit: anti‐infection (acyclovir), plasma exchange, liver protection (reduced glutathione, ursodeoxycholic acid), nutritional support, and other treatments after admission. The boy was transferred from PICU to the general ward of the gastroenterology department on the Day 11, and was discharged after 7 days of subsequent therapy. So far, he has no symptoms. His liver function is normal.

## 4 DISCUSSION

Acute liver failure (ALF) in children is a clinical syndrome that can involve multiple organs and systems. It is mainly manifested by rapidly progressing liver function damage and abnormal blood coagulation, with or without hepatic encephalopathy. ALF can occur in a child without a clear history of chronic liver disease.^7^ The case fatality rate can reach 44% ~67% in the children with poor prognosis.^7^, ^8^, ^9^ The causes of ALF include infections, drug poisons, genetic metabolic diseases, vascular factors, autoimmune factors, malignant tumors, etc.^10^ The etiology of ALF in children varies depending upon regions and ages. In Europe and the United States, acetaminophen is the main cause. While in developing countries, the cause of infection is more common, along with 38%–49% of children's ALF etiology that is unknown.^9^ Possible causes of unexplained liver failure include rare viral infections, such as hidden HBV infection, human herpesvirus‐6 (HHV‐6), herpes simplex virus (HSV), varicella zone Herpes zoster virus (VZV), human parvovirus B19, transfusion‐transmitted virus, hepatitis G virus (HGV), and Reye Syndrome, autoimmune hepatitis (AIH), rare parasitic infections, environmental toxic effects. At present, only a few samples or case studies have been reported on ALF caused by the aforementioned causes.^11^, ^12^ HHV‐6 is divided into HHV‐6A type and HHV‐6B type, while the latter is common. HHV‐6B can be spread through saliva, droplets, close contact, blood transfusion, and organ transplantation. HHV‐6 infection is more common among the population, and the most common cause of parascarlatina. The positive rate of HHV‐6 antibody in three‐year‐old infants is over 95% in Guangdong Province, China. Because viral replication quantity are few and process is slow, apart from immunodeficiency (such as congenital immunodeficiency, AIDS, and long‐term use of immunosuppressive agents), most people infected with HHV‐6 are asymptomatic throughout life, that is, HHV‐6 lifelong carrier. Previous studies showed that HHV‐6 can cause liver failure, encephalitis, epilepsy, Hashimoto thyroiditis. Early clinical manifestations of HHV‐6 infection are fever and other non‐specific symptoms, such as rash, leukopenia, and thrombocytopenia. HHV‐6 infection is usually acquired very early in life, between 6 months and 2 years of age, following the loss of protective maternal antibodies,^13^ which is consistent with this case. We use mNGS method to detect HHV6‐B. In addition, the monocytes continued to increase at the peak of the disease. Blood smear heteromorphic lymphocyte were 12% in the second day after admission. T‐cell, B‐cell, and NK‐cell all decreased. However, the patient had been in good health. Therefore, the possibility of secondary immune deficiency caused by virus infection was considered. In view of the specific role of the protein encoded by HHV‐6, they act as analogs of cellular chemokines and are believed to promote viral growth, viral dissemination, and/or escape from the immune response.^14^ It was reported that a woman with normal immunity suffered from liver failure due to infection with HHV‐6.^15^ In the case, the boy had no special disease history or had no immunosuppressive agents. If HHV‐6B infection was the cause of the disease, was there any incentive? The patient's mother was a nurse. In order to prevent the abuse of drugs to the boy with fever, only ibuprofen suspension drops and amoxicillin were taken. It was REPORTED THAT AMOXICILLIN IS CAPable of directly stimulating HHV‐6 replication, which induces ALF [12].^16^ But under what circumstances can amoxicillin induce HHV‐6 replication? It is unclear after all HHV‐6 is widely present in the population. Amoxicillin is also a commonly used antibacterial drug. Acute liver failure caused by amoxicillin is rare.

The mutation in *NBAS* gene can cause autosomal recessive diseases. The study found that mutation in *NBAS* gene can cause some complex diseases with a wide range of clinical manifestations. SOPH syndrome (short stature, Pelger–Huёt cell deformity, optic nerve atrophy, and no obvious liver failure) and ILFS‐2 are common.^17^, ^18^ The most common *NBAS* mutation site is c. 5741G>A homozygous mutation. In our case, there is a homozygous missense mutation c.3596G>A (p. Cys1199Tyr). This mutation is manifested as acute liver failure (Fever, vomiting, coma, increased liver enzymes, jaundice, abnormal blood coagulation, hyperammonemia, hypoglycemia, and encephalopathy). The specific mechanism is not yet clear. It may be due to the increased sensitivity of *NBAS*‐deficient patients to fever, promoting inflammation factors cause liver inflammation and cell lysis.^19^ Currently, the ILFS‐2 patients reported in Chinese children all have c. 3596G>A mutation site, suggesting that this site may be a Chinese hot spot.^5^, ^6^ Patients with *NBAS* mutations had febrile disease before each liver function injury. After 24–72 h of fever, ALT and AST activities were significantly increased.^6^ Mutations in *NBAS* are delineated as a previously unknown cause of acute liver failure with onset in childhood triggered by febrile infections.^20^ The exact mechanism of liver failure in *NBAS* deficiency is unclear and the physiologic function of *NBAS* remains to be further investigated.^19^ The diagnosis of diseases caused by mutations in *NBAS* gene is complicated and mainly depends on the clinical manifestations and genetic testing.

The treatment of *NBAS* gene mutation disease is currently not reported yet. In this case, timely plasma exchange treatment plays a key role in the recovery of the patient. Other treatment is routine symptomatic support treatment. Individualized treatment should be carried out according to the patient's condition. However, the prognosis and quality of life of the patient are not clear, so regular follow‐up and further study should be performed.

## 5 CONCLUSION

In summary, we believe that the boy's acute liver failure was induced by oral amoxicillin and homozygous mutation in *NBAS* gene after HHV‐6B infection. c. 3596 G>A (p. Cys1199Tyr) homozygous variation was different from the compound heterozygous of heterozygous point and other mutations reported in the literatures. Therefore, children with unexplained fever and liver failure should be inquired about the medical history carefully, especially the history of medication, and conduct NGS and gene sequencing.

## CONFLICT OF INTEREST

The author(s) declared no potential conflicts of interest with respect to the research, authorship, and/or publication of this article.

## AUTHOR CONTRIBUTIONS

YC conceived the idea and wrote the initial draft of the manuscript. HX and YC critically reviewed and revised the overall content of the manuscript. All the authors were involved in the care of the patient. All authors read and approved the final manuscript.

## CONSENT FOR PUBLICATION

Written informed consent was obtained from the parents of this patient. A copy of the written consent is available for review by request.

## Data Availability

All data generated and/or analyzed during this study are included in this published article.
